# Risk Stratification Using Multivariable Fractional Polynomials in Diffuse Large B-Cell Lymphoma

**DOI:** 10.3389/fonc.2020.00329

**Published:** 2020-03-11

**Authors:** Jin Roh, Jiwon Jung, Yourim Lee, So-Woon Kim, Hyo-Kyung Pak, A-Neum Lee, Junho Lee, Jaehyeong Cho, Hyungwoo Cho, Dok Hyun Yoon, Rae Woong Park, Jooryung Huh, Heung-Bum Oh, Chan-Sik Park

**Affiliations:** ^1^Department of Pathology, Ajou University School of Medicine, Suwon, South Korea; ^2^Department of Pathology, Asan Medical Center, University of Ulsan College of Medicine, Seoul, South Korea; ^3^Asan Medical Center, Asan Institute for Life Science, University of Ulsan College of Medicine, Seoul, South Korea; ^4^Department of Biomedical Informatics, Ajou University School of Medicine, Suwon, South Korea; ^5^Department of Biomedical Sciences, University of Ulsan College of Medicine, Asan Medical Center, Seoul, South Korea; ^6^Convergence Medicine Research Center, Asan Medical Center, Seoul, South Korea; ^7^Department of Biomedical Science, Ajou University Graduate School of Medicine, Suwon, South Korea; ^8^Department of Oncology, Asan Medical Center, University of Ulsan College of Medicine, Seoul, South Korea; ^9^Department of Laboratory Medicine, Asan Medical Center, University of Ulsan College of Medicine, Seoul, South Korea

**Keywords:** diffuse large B-cell lymphoma, prognosis, multivariable fractional polynomial, risk stratification, prognostic model

## Abstract

The risk stratification of diffuse large B-cell lymphoma (DLBCL) is crucial. The International Prognostic Index, the most commonly used and the traditional risk stratification system, is composed of fixed and artificially dichotomized attributes. We aimed to develop a novel prognostic model that allows the incorporation of up-to-date attributes comprehensively without information loss. We analyzed 204 patients with primary DLBCL who were uniformly treated with R-CHOP (rituximab, cyclophosphamide, doxorubicin, vincristine, and prednisone) from 2007 to 2012 at Asan Medical Center. Using the multivariable fractional polynomial (MFP) method and bootstrap resampling, we selected the variables of significance and the best fitted functional form in fractional polynomials. Age, serum β2-microglobulin, serum lactate dehydrogenase, and BCL2 expression were selected as significant variables in predicting overall survival (OS), while age was excluded in predicting 2-years event-free survival. The prognostic score calculated by the MFP model effectively classifies patients into four risk groups with 5-years OS of 89.91% (low risk), 81.21% (low-intermediate risk), 66.40% (high-intermediate risk), and 37.89% (high risk). We suggest a new prognostic model that is simple and flexible. By using the MFP method, we can incorporate various clinicopathologic factors into a risk stratification system without arbitrary dichotomization.

## 1. Introduction

Diffuse large B-cell lymphoma (DLBCL) is an aggressive and the most prevalent subtype of non-Hodgkin lymphoma (NHL) (1, 2). Despite the improvement of overall outcomes with up-front immunochemotherapy (rituximab, cyclophosphamide, doxorubicin, vincristine, and prednisone [R-CHOP]), ~40% of patients with DLBCL fail to achieve remission and ultimately succumb to death (3–5). One reason for treatment failure is the limitations of current prognostication systems for DLBCL. Since 1993, the International Prognostic Index (IPI) has been the most commonly used clinical tool for the risk stratification of patients with DLBCL (6). It considers age (>60 years), the Ann Arbor Stage (III/IV), serum lactate dehydrogenase (LDH) levels (>upper limit of normal), performance status [Eastern Cooperative Oncology Group (ECOG) >1], and the number of extranodal involvements (>1). However, the IPI does not completely predict the prognosis of patients and also narrows the outcome differences between the IPI risk groups (7). In addition, the risk stratification system solely based on clinical factors does not reflect the fundamental biological properties of the tumors. Recently, with the advancement of research on the pathophysiology of DLBCL, many biological and molecular features, such as the cell of origin (COO), BCL2 and MYC status, and genetic alteration, have been discovered to be associated with poor prognosis (8–13). Additionally, new therapeutic agents, including immune checkpoint blockers, are undergoing clinical trials to overcome the limitation of current R-CHOP therapy (14). Therefore, there is an impending unmet medical need to develop a risk stratification system that allows incorporation of the latest clinical and biological attributes comprehensively.

One limitation of the current IPI is that attributes that originally had continuous values are dichotomized by using the artificially assigned thresholds. To build prognostic models, most studies have included continuous predictors as categorized forms with their arbitrary optimal cut-off points. Although there has been constant criticism of this approach (15–17), categorization is widespread in clinical studies (18). One of the perceived advantages of categorization is that it is easy to apply to clinical practice that determines the diagnostic or therapeutic procedures. However, finding an “optimal” cut-off point is virtually infeasible; it has been reported that neither Kaplan–Meier nor the receiver operating characteristic (ROC) methods can be relied upon to represent a true biological threshold in prognosis (19). Fundamentally, categorization induces the inevitable loss of information and statistical power and may increase the probability of false-positive results (20–22).

To overcome these pitfalls of dichotomization, Royston et al. developed the multivariable fractional polynomial (MFP) approach to build models from multiple predictors with a combination of continuous and categorical variables (23, 24). In handling the continuous variable, a logistic regression model presumes a linear relationship between covariates and a response variable in the logit scale. However, various functional forms of covariates which incorporate non-linear relationships should also be considered (25). For that reason, the MFP model uses possible transformed predictors with various powers and performs closed test to select significant predictors. In brief, the MFP method can be explained in two main concepts: backward elimination among all possible predictors and selection of an fractional polynomial (FP) function to incorporate non-linear relationship of continuous variables (26). This allows us the determination of whether an explanatory variable is important and the return of its optimal functional form among the possible combinations of fractional polynomials (FP) (27). In addition, using the prognostic index which is the combination of selected variables with weights taken from the Cox model can explain the relative hazard of the patient with certain predictor values.

In this study, we developed a new risk stratification model for DLBCL using the MFP method. Among the various clinical and biological factors related to DLBCL, statistically significant variables were selected while retaining the properties of the continuous variables. We selected suitable FP functions among selected variables to build a parsimonious and medically consistent final model. The model stability was also investigated using the bootstrap assessment. The final survival model was formed by fitting a Cox model with finally selected covariates. Using this MFP method, we suggest a clinically feasible and flexible model that can comprehensively allow for continuously updating clinical and biological attributes.

## 2. Materials and Methods

### 2.1. Patients and Clinicopathologic Information

For the analysis, clinicopathologic information was retrospectively collected from patients with primary DLBCL diagnosed between 2007 and 2012 at Asan Medical Center. A total of 204 cases were comprehensively reviewed, and diagnoses were confirmed by two expert hematopathologists (RJ and CSP) according to the 2016 WHO Classification of Tumors of Haematopoietic and Lymphoid Tissues (2). All patients underwent the standard staging procedures and were treated with R-CHOP. Patients with primary central nervous system lymphoma and patients who were initially treated with other treatments rather than R-CHOP were excluded. Clinicopathological information known to be associated with the prognosis of DLBCL was meticulously obtained from medical records including sex, age, body mass index (BMI) (28), status of concurrent hepatitis B virus infection (29), levels of serum LDH and B2M (30), hemoglobin (Hb) levels (31), and baseline peripheral absolute neutrophil, lymphocyte, and monocyte counts (32), Ann Arbor stage, ECOG performance status, presence of B symptoms, involvement of two or more extranodal sites, and COO. The COO was determined using immunohistochemistry (IHC) according to the Hans classification at the time of diagnosis (33).

Among the clinicopathological variables, B2M contained 11 missing values, which accounted for 5.39% of the total. These missing values were imputed by multiple imputations using the Amelia package in R which is known to be effective for handling large numbers of missing data (34). This study was approved by the institutional review board (No. 2015-0720).

### 2.2. Quantitative Analysis of Biomarker Expression

We selected BCL2 and MYC as candidates for predictors because numerous studies on DLBCL have reported their association with poor prognosis (9, 35, 36). Although the proportions of 40 and 50% were generally considered as cut-off points for positivity in MYC and BCL2 IHC, respectively (2), it was mostly dependent on visual observation, and there have been controversies regarding various proportional cut-off points (36, 37). Therefore, we employed a digital quantitative acquisition and analysis for the objective measurements of BCL2 and MYC expressions without dichotomization.

The quantitative analyses of BCL2 and MYC expressions were performed using multiplex immunofluorescence (IF) labeling and the Automated Quantitative Analysis (AQUA) scoring method to minimize interobserver variation. Multiplex IF labeling with tyramide signal amplification was performed using the Opal IHC Kit (NEL8100KT, PerkinElmer) according to the manufacturer's instructions. To analyze tumor-specific biomarker expression, tumor cells were selected using CD20 expression. The tumor-specific quantitative immunofluorescence (QIF) score representing protein expression on a cell was calculated using the AQUA scoring method. The QIF scores for BCL2 and MYC were calculated as the signal intensities of each biomarker in the target compartment divided by the pixel area of the target compartment (38) (Figure S1). Details on multiplex IF labeling and QIF scoring are provided in the Supplementary Material.

### 2.3. Modeling With Multivariable Fractional Polynomials

Using the MFP algorithm, which variables are significant and what functional form to take is determined through iterative fashion. At first, the complexity of the functional form for continuous variables and a nominal *P*-value for the inclusion of variables were determined. The maximum degree of FP for each continuous variable was set at two to prevent the formation of overly complex model, and the nominal significance level for testing variables and functions was set at the conventional 0.05 level (39). FP of a certain degree contains various terms, depending on the number of powers allowed. By convention, powers are selected from the collection (−2, −1, −0.5, 0, 0.5, 1, 2, 3), where 0 indicates the log transformation. Repeated power indicates powers of log(*X*). For example, an FP2 with powers (−1, −1) is of the form $\beta_{0}\;+\;\beta_{1}X^{-1}\;+\;\beta_{2}X^{-1}\log\left(X\right)$. All categorical variables are not subject to FP transformation and are modeled with one degree of freedom. Among these categorical variables and FP-transformed continuous variables, significant variables were selected by using backward elimination.

To reduce the risk of overfitting and find a stable multivariable model, bootstrap resampling was performed (40, 41). After one thousand bootstrap replications, variables for the final model were selected according to the resulting bootstrap inclusion fractions (BIF). BIF are defined as the proportion of bootstrap replications in which a given variable or type of function selected by MFP (41). To obtain a stable and interpretable model, variables with BIF >60% were selected for the final model. The final model in the survival analysis aimed to produce a prognostic index which is a weighted combination of the predictors with weights (regression coefficients) taken from the Cox model. The prognostic index value for a given individual summarizes the relative hazard of that person with respect to the control population.

### 2.4. Statistics

We verified the performance of the fitted Cox model using the resampling model calibration of the rms and time-dependent receiver operating characteristic (ROC) curve of the survivalROC packages, respectively. Survival curves were plotted using the Kaplan–Meier method and the log-rank test was used to analyze the statistical differences between survival curves. *P*-values in the univariate analysis were adjusted using the Benjamini-Hochberg procedure considering multiple comparison testing. The nomogram for predicting overall survival with the calculated prognostic score was drawn using the rms package (42). All statistical calculations including MFP modeling were conducted using R version 3.4.0. (R Foundation for Statistical Computing, https://www.R-project.org/).

### 2.5. Outcomes

Overall survival (OS) was defined as the time from diagnosis until death as a result of any cause. Event-free survival (EFS) was defined as the time from diagnosis until relapse or progression, unplanned re-treatment of lymphoma after initial immunochemotherapy, or death as a result of any cause. OS and EFS time were measured in months. Outcome indicators with given cut-off points (ie, EFS at 24 months [EFS24]) were defined based on the outcome status at each cut-off point from the date of the diagnosis (43).

## 3. Results

### 3.1. Patient Characteristics

For the 204 patients, OS60 and EFS24 were 69.5 and 71.5%, respectively, with a median follow up of 59 months. The clinicopathological characteristics of the patients are summarized in Table 1. Seven categorical (Sex, B symptom, COO, ECOG, Ann Arbor stage, presence of HBsAg, and extranodal involvement) and ten continuous (age, B2M, BMI, ANC, ALC, AMC, Hb, LDH, BCL2 QIF score, and MYC QIF score) variables were considered as candidates for selection. ECOG and Ann Arbor stage were classified into two groups: low (0–1)/high (2–5) ECOG and low (1–2)/high (3–4) Ann Arbor stage. Results of univariate analysis for OS and EFS24 were also described in Table 1. Fluorescence *in situ* hybridization (FISH) results for *BCL2* and *MYC* were available in 50 (24.51%) patients. Of those 50 patients, only one patient (2.00%) showed rearrangements of both *BCL2* and *MYC*. All potential explanatory covariates were applied to the initial cycle, because the MFP method uses backward elimination for variable selection. Initially, the Spearman correlation matrix was examined to investigate the dependence between variables (Figure S2). Moderately strong positive correlations were noted between the serum LDH and B2M levels (correlation coefficient, 0.57). The Ann Arbor Stage and extranodal involvement showed a strong positive correlation (correlation coefficient, 0.66). There were no significant correlations among other variables. These results were later considered in developing a parsimonious multivariable model.

**Table 1:** Baseline characteristics of patients.

**Characteristics**		***N* (total = 204)**	**%**	**OS**		**2 years EFS**	
				**HR (95% CI)**	***P*-value**	**HR (95% CI)**	***P*-value**
Age (years)	Median	59					
	Range	20–82		1.0468 (1.02–1.07)	0.0003	1.0247 (1.00–1.05)	0.0582
Sex	Male	117	57.4	1.3362 (0.80–2.24)	0.3245	1.3568 (0.80–2.29)	0.3530
B symptom	Present	56	27.5	1.6901 (1.01–2.83)	0.0919	1.4155 (0.83–2.42)	0.3068
β2-microglobulin (mg/L)	Median	2.1					
	Range	0.2–15.1		1.2040 (1.10–1.31)	0.0002	1.1984 (1.10–1.31)	0.0004
BMI	Median	23.5					
	Range	17.2–33.6		0.9479 (0.87–1.03)	0.2827	0.9755 (0.90–1.06)	0.6316
ANC (/μl)	Median	4130					
	Range	20–16,450		1.000 (1.00–1.00)	0.870	1.000 (1.00–1.00)	0.4916
ALC (/μl)	Median	1612					
	Range	0–11,210		0.9996 (0.99–1.00)	0.0929	0.9996 (0.99–1.00)	0.1021
AMC (/μl)	Median	523					
	Range	0–6,000		1.0001 (1.00–1.00)	0.5883	1.000 (1.00–1.00)	0.8858
COO	Non-GCB	139	68.1	1.4304 (0.80–2.56)	0.2927	1.2432 (0.70–2.20)	0.5470
ECOG	High (2–5)	21	10.3	4.1723 (2.32–7.51)	<0.0001	3.4683 (1.90–6.32)	0.0004
Ann Arbor Stage	High (3–4)	116	56.7	1.7883 (1.04–3.07)	0.0789	1.697 (0.99–2.92)	0.1021
Extranodal (>1)		88	43.1	2.028 (1.22–3.37)	0.0224	1.834 (1.10–3.06)	0.0582
Hb (g/dl)	Median	12.0					
	Range	7.1–16.2		0.8918 (0.79–1.01)	0.1166	0.8585 (0.76–0.98)	0.0582
LDH (IU/L)	Median	235.5					
	Range	93–7,131		1.0002 (1.00–1.00)	0.1011	1.0002 (1.00–1.00)	0.1021
BCL2 QIF	Median	32.5					
	Range	0–100		1.0205 (1.01–1.03)	0.0080	1.0231 (1.01–1.04)	0.0031
MYC QIF	Median	36.2					
	Range	0–100		1.0168 (1.00–1.03)	0.0463	1.0195 (1.01–1.03)	0.0160

*BMI, body mass index; ANC, absolute neutrophil count; ALC, absolute lymphocyte count; AMC, absolute monocyte count; COO, cell of origin; ECOG, Eastern Cooperative Oncology Group; LDH, lactate dehydrogenage; QIF, quantitative immunofluorescence; OS, overall survival; EFS, event-free survival; P-values were adjusted using the Benjamini-Hochberg procedure*.

### 3.2. Building an MFP Model and Bootstrap Resampling

An initial MFP model was built for the entire cohort with variables described above at the conventional 5% of significance level (*p* <0.05) (39). For OS, age (*p* = 0.003) and ECOG group (*p* = 0.001) were selected as statistically significant variables in the model. For EFS24, B2M (*p* = 0.001) and BCL2 QIF score (*p* = 0.031) were selected in the model. Age and BCL2 QIF score were best fitted in the model when they were transformed as first-degree FP (FP1) with power 1. B2M was best fitted when it was transformed as FP1 with power −0.5 (Table 2). These results suggest that age, ECOG group, and BCL2 QIF score were the most important predictors in the initial MFP model. However, this model was difficult to consider as stable because the model targeted a single data set.

**Table 2:** Summary of the MFP algorithm applied to the DLBCL patient data set.

		**OS**			**EFS24**		
**Predictor**		**In/out of model**	***P*-value^***a***^**	**FP**	**In/out of model**	***P*-value^***a***^**	**FP**
**BINARY**							
	Sex	Out	0.415		Out	0.277	
	B symptom	Out	0.414		Out	0.760	
	COO	Out	0.408		Out	0.397	
	ECOG group	In	0.001	N/A	Out	0.056	
	Stage group	Out	0.276		Out	0.578	
	Extranodal involvement	Out	0.102		Out	0.440	
**CONTINUOUS**							
	Age	In	0.003	FP1(1)	Out	0.595	
	β2-microglobulin	Out	0.088		In	0.001	FP1 (−0.5)
	BMI	Out	0.397		Out	0.829	
	ANC	Out	0.314		Out	0.311	
	ALC	Out	0.435		Out	0.224	
	AMC	Out	0.514		Out	0.450	
	Hb	Out	0.485		Out	0.824	
	LDH	Out	0.195		Out	0.388	
	BCL2 QIF	Out	0.050		In	0.031	FP1 (1)
	MYC QIF	Out	0.807		Out	0.678	

a *At the final cycle of the MFP algorithm. COO, cell of origin; ECOG, Eastern Cooperative Oncology Group; BMI, body mass index; ANC, absolute neutrophil count; ALC, absolute lymphocyte count; AMC, absolute monocyte count; Hb, hemoglobin; LDH, lactate dehydrogenase; QIF, quantitative immunofluorescence; MFP, multivariable fractional polynomial; FP, fractional polynomial; DLBCL, diffuse large B-cell lymphoma; OS, overall survival; EFS24, 2-years event-free survival*.

The stability of the established model was investigated by bootstrap resampling (Table 3). The same algorithm was applied to 1,000 bootstrap samples for OS and EFS24, respectively. Some algorithms built with bootstrap samples failed to converge. However, it was presumed that this did not affect the results because the model was successfully developed in over 90% of the replications. Overall, 958 and 966 MFP models with replications were built for OS and EFS24, respectively. BIF and frequencies for selected power are described in Table 3. Although the ECOG group was selected in the initial model, the inclusion proportions were only 47.60 and 36.54% in OS and EFS24, respectively. BIF for age was 88.94% in OS but only 40.68% in EFS24. In contrast, some of the variables that were not included in the initial model were frequently included in the replications. In addition, 60% of BIF was selected as a cut-off point for the importance of a variable considering the model complexity and clinical significance. The variables with more than 60% of BIF were as follows: age (88.94%), B2M (78.39%), LDH (60.86%), and BCL2 QIF score (64.09%) for OS; B2M (76.92%), LDH (65.42%), and BCL2 QIF score (66.25%) for EFS24. Between FP1 and FP2 transformations of each variable, a polynomial transformation was chosen that was selected more frequently in the model. Because second-degree FP consists of FP1 and additional polynomials, the BIF for FP1 contains that of FP2. Therefore, to determine the BIF of FP1 alone, the BIF of FP2 must be subtracted from that of FP1. Because the FP2 BIF for LDH was 41.44% out of 60.86% of the entire FP1 BIF, LDH was mainly selected with FP2 transformation in both OS and EFS24. The mainly selected polynomial and its power for each variable were as follows: age FP1 (1), LDH FP2 (3, 3), B2M FP1 (0), and BCL2 QIF score FP1 (1).

**Table 3:** Numbers and percentages of selected powers from 1,000 bootstrap replications.

**Variable**	**Power**	**Included (OS) (*****n*** **=** **958)**		**Included (EFS24) (*****n*** **=** **966)**	
		**BIF (*n*)**	**BIF (%)**	**BIF (*n*)**	**BIF (%)**
Age	p1	852	88.94	393	40.68
	p2	328	34.24	183	18.94
Sex		232	24.22	331	34.27
B symptom		103	10.75	145	15.01
β2-microglobulin	p1	751	78.39	743	76.92
	p2	304	31.73	306	31.68
BMI	p1	241	25.16	216	22.36
	p2	39	4.07	124	12.84
ANC	p1	566	59.08	527	54.55
	p2	297	31.00	304	31.47
ALC	p1	543	56.68	491	50.83
	p2	374	39.04	332	34.37
AMC	p1	201	20.98	491	50.83
	p2	90	9.39	224	23.19
COO		113	11.80	135	13.98
ECOG low/high		456	47.60	353	36.54
Stage low/high		151	15.76	156	16.15
Extranodal (>1)		159	16.60	149	15.42
Hb	p1	192	20.04	139	14.39
	p2	132	13.78	29	3.00
HBsAg		254	26.51	105	10.87
LDH	p1	583	60.86	632	65.42
	p2	397	41.44	396	40.99
BCL2 QIF	p1	614	64.09	640	66.25
	p2	51	5.32	43	4.45
MYC QIF	p1	217	22.65	298	30.85
	p2	94	9.81	118	12.22

*BIF, bootstrap inclusion fractions; BMI, body mass index; ANC, absolute neutrophil count; ALC, absolute lymphocyte count; AMC, absolute monocyte count; COO, cell of origin; ECOG, Eastern Cooperative Oncology Group; LDH, lactate dehydrogenage; Hb, hemoglobin; QIF, quantitative immunofluorescence; OS, overall survival; EFS24, 2-years event-free survival*.

### 3.3. Final Model and Prognostic Index

To build a parsimonious model, it is necessary to review each selected variable and simplify unnecessary complex transformations. As a result of the preceding analysis, a non-monotonic second-degree FP was chosen for LDH with powers (3, 3). Full curves of the LDH-component of the log-hazard-ratio (HR) from both models with FP1 and FP2 were compared (Figure S3). The model with the first-degree FP for LDH was adjusted to 0 at 0.0008 which was 263.62 in raw data. In contrast, the second-degree FP for LDH was adjusted to 0 at −0.00611 which was 92.80 in raw data. Considering the general clinical knowledge regarding 250 IU/l as a cutoff value for increased serum LDH, the first-degree FP for serum LDH was selected for to the final model. In addition, dependence for inclusion between B2M and LDH was also analyzed because of their positive correlation (Figure S2). In all 958 replications, both LDH and B2M were selected in 463 replications (48.32%). When LDH was omitted, B2M was included in 288 replications (30.06%), and LDH was included in 120 replications (12.53%) when B2M was omitted (Table S1). This result supports that both variables of B2M and LDH should be included in the final model.

With the final model, prognostic scores for OS and EFS24 were calculated using the Cox model. The formulae for OS and EFS24 are as follows:

$$\begin{array}{l} \left[OS\right]\;Prognostic\;index\;=\;3.78\;\times \;Age\;+\;0.0022\;\times \;{\left(\frac{LDH}{1000}\right)}^{3}\\ \;+\;0.096\;\times \;B2M\;+\;1.8\;\times \;\frac{BCL2}{100}\\ \left[EFS24\right]\;Prognostic\;index\;=\;0.0011\;\times \;{\left(\frac{LDH}{1000}\right)}^{3}\\ \;+\;0.153\;\times \;B2M\;+\;2.11\;\times \;\frac{BCL2}{100} \end{array}$$

The performance of the fitted Cox model was assessed in terms of calibration and discrimination. In resampling model calibration, the mean absolute calibration error for OS and EFS were 5.9 and 2.9%, respectively (Figures S3A,B). For evaluating discrimination, time-dependent ROC curves were plotted (Figures S3C,D). After the last observed date for OS60 and EFS24, the area under the curve (AUC) stabilized at 0.68 for OS60 and 0.71 for EFS24, without decay of the performance.

**Figure 1:**
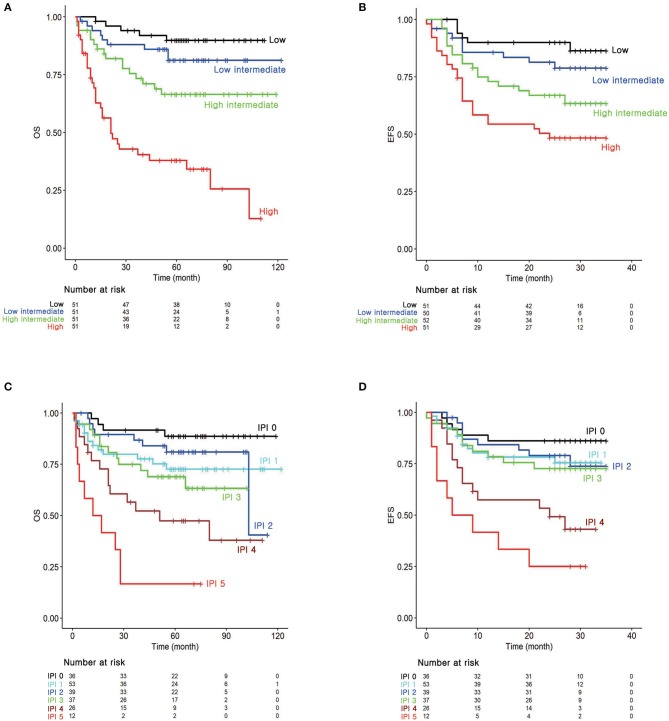
Survival analysis according to the risk groups. Kaplan–Meier survival curves for **(A)** overall survival (OS) and **(B)** 2-years event-free survival (EFS24) according to the prognostic index from the MFP model. **(C)** Kaplan–Meier survival curves for OS and **(D)** EFS24 according to the IPI.

Kaplan–Meier survival curves were depicted by applying temporary cutoff values to the prognostic score at the 25th, 50th, and 75th quantiles (Figures 1A,B). Patient groups were named as low, low-intermediate, high-intermediate, and high-risk groups from the low-scoring order. OS60 for low (*n* = 51), low-intermediate (*n* = 51), high-intermediate (*n* = 51), and high-risk (*n* = 51) patients were 89.81, 81.21, 66.40, and 37.89%, respectively. EFS24 for patients in each risk group were 89.96, 79.70, 68.26, and 48.33%, respectively. These survival curves classified patients as efficiently as those of conventional IPI (Figures 1C,D). Notably, log-HR values for OS and EFS24 using prognostic scores showed a continuously increasing tendency, while IPI showed non-continuous and inconsistent trends (Figure S4). Survival curves and log-HR values were also plotted with IPI risk group (Figure S6). IPI risk group was classified as low (IPI 0–1), low-intermediate (IPI 2), high-intermediate (IPI 3), and high (IPI 4–5). The results were similar to those with IPI. This promising result implies that the prognostic score calculated from the MFP model efficiently classifies the prognosis of patients with DLBCL.

## 4. Discussion

Recent advances in molecular pathobiology confirmed that DLBCL is highly heterogeneous both genetically and biologically (12, 44). Proper risk stratification and patient selection are required for both clinical practice and new drug development (45). The current risk stratification systems for DLBCL, such as IPI, have limitations in incorporating newly discovered tumor-intrinsic prognostic factors. Moreover, determining optimal cut-off points for these pathobiological prognostic factors is highly difficult as readouts of most biological molecules are continuous variables. Several studies have shown that the MFP approach provides an improved predictive ability in analyzing medical data that consists of various categorical and continuous variables (25, 46, 47). In this study, we developed a prognostic model using the MFP method in patients with DLBCL. By using this method, continuous variables could be used as they were without dichotomization, which minimized information loss. In addition, this model produced a prognostic score for each patient as a continuous variable, unlike that in the traditional methods. As a result, age, serum LDH, serum B2M, and BCL2 QIF score were selected to calculate prognostic score for OS. Serum LDH, serum B2M, and BCL2 QIF score were selected to calculate the prognostic score for EFS24. The higher the prognostic score of the patient, the worse was the prognosis of the patient. Risk groups based on the prognostic score from the MFP model were well-separated by their prognosis.

Attributes associated with the biological characteristics of tumors, such as serum LDH and B2M, were selected in the model for short-term outcomes (EFS24). Notably, age was additionally selected in the prognostic model for the long-term outcome (OS) compared to the prognostic model for the short-term outcome. These results imply the overall status of a patient may play an important role in their long-term prognosis. The serum LDH level is included in the current IPI and represents the tumor burden. In addition to serum LDH, serum B2M was also selected in the prognostic model for both OS and EFS24. The serum B2M levels in a patient with lymphoma, similar to the serum LDH levels, have been widely accepted as directly related to tumor burden (48). However, serum B2M levels also imply involvement of the immune reaction to the tumor because it is a component of major histocompatibility complex (MHC I) (49, 50). Based on these grounds, several studies have shown the poor prognostic effect of serum B2M in DLBCL (30, 51, 52). However, these results are controversial, and most of these studies have shown difficulties in determining the universal optimal cut-off point. The prognostic effect of serum B2M in DLBCL as a continuous value was confirmed without determining a specific cut-off point.

Using the current risk stratification system of hematologic malignancy, it is difficult to comprehensively predict the prognosis of patients with DLBCL. A major limitation of the IPI system is that it does not allow the incorporation of newly discovered prognostic factors, most notably BCL2 and MYC. BCL2 and/or MYC expression in DLBCL is associated with aggressive behavior and poor prognosis (9, 35, 36, 53). However, the proportional cut-off points of their expressions are still arbitrary, and a recent study has shown the importance of intensity of BCL2 expression as well as its extent (36). To overcome this limitation and produce an objective, the continuous variable for measuring protein expression, multiplex IF for BCL2 and MYC with tumor marker CD20 was exploited. Fluorescence is more linear and has a wider dynamic range compared to those of chromogenic immunohistochemistry (54), which allows object acquisition of the expression amount of each protein. As a result, the BCL2 QIF score was selected as a predictor of poor prognosis, which is consistent with previous findings. However, MYC expression was not selected in the current prognostic model. Of note, COO was not found to be an important predictor and this result is consistent with the existing controversies on the prognostic importance of COO (55). These results suggest that our prognostic model is flexible enough to incorporate biomarkers of DLBCL into a single risk stratification system and is consistent with the results of other previous studies. It can be also used to improve the prognostic ability of the existing prognostic model by adding continuously updating new information (56).

The limitation of this study is the relatively small sample size and event number. Also, the lack of external validation can be another limitation. To overcome these limitation and establish a stable prognostic model, we performed bootstrap resampling method. Although we verified the model using the internal validation, further external validation study may potentially provide better insight for performance of the model. Regarding the relatively small event number, the final number of variables for a Cox regression model was two or three. Considering the suggestions of previous simulation studies that minimum events per variable values of between 5 and 20 were needed for reliable results, the final number of covariates of this study seems to be reasonable (57).

In this study, we suggest a new prognostic model using the MFP method in DLBCL, which allows the flexible incorporation of variable clinicopathological factors into a single risk stratification system. It is a simple and interpretable model consisting of only objectively quantified measurements and incorporates non-linear relationships. The model also presents a continuous prognostic score in each patient that can provide enormous flexibility in classifying risk groups. In the clinical practice, this prognostic score can be used in the form of a nomogram predicting patients' survival (Figure S7). The MFP method has been studied in some solid cancers including breast cancer and renal cell carcinoma (39, 56, 58). Prognostic modeling using the MFP method with full information resulted in better performance in previous studies. Most notably, this is the first study to our knowledge to investigate the effectiveness of MFP methods in hematological malignancies. Further studies using a large patient population will increase the generalizability of this method.

## Data Availability Statement

The data sets used and/or analyzed during the current study are available from the corresponding author on reasonable request.

## Ethics Statement

The studies involving human participants were reviewed and approved by Institutional Review Board of Asan Medical Center (No. 2015-0720). Written informed consent for participation was not required for this study in accordance with the national legislation and the institutional requirements.

## Author Contributions

C-SP and H-BO contributed to conceptualization, study design, manuscript preparation, and editing. JR contributed to drafting the manuscript and coordinating the data collection and analysis. YL contributed to data analysis. JJ, JC, JL, and RP contributed to the interpretation of the statistical results and manuscript editing. A-NL contributed to performing the immunofluorescence assay. H-KP and S-WK contributed to data collection. HC, DY, and JH contributed to the interpretation of clinicopathological data. All authors approved the final submitted manuscript.

### Conflict of Interest

The authors declare that the research was conducted in the absence of any commercial or financial relationships that could be construed as a potential conflict of interest.
