# Supplementary material for: Risk Stratification Using Multivariable Fractional Polynomials in Diffuse Large B-Cell Lymphoma
Source: Front Oncol. 2020 Mar 11;10:329. doi: 10.3389/fonc.2020.00329 (PMC7078241; doi:10.3389/fonc.2020.00329)
Supplement: Supplementary file 1 [file Data_Sheet_1.PDF]

Supplementary Material

1 SUPPLEMENTARY MATERIALS AND METHODS

1.1 Multiplex IF and AQUA scoring

Briefly, 4μm-thick sections deparaffinized in xylene and dehydrated in graded ethanols. Antigen retrieval was performed in citrate buffer (pH 6.0) with microwave heating. Primary antibodies were as follows: CD20 (clone L26; Dako), CD3 (polyclonal (cat. A0452); Dako), BCL2 (clone 124; Dako), and MYC (clone Y69; Abcam). Envision+ poly-HRP-anti-mouse and Envision+ poly-HRP-anti-rabbit (Dako) were used as secondary antibodies. Staining was optimized by performing a duplex (CD20 – Opal 650 and BCL2 – Opal 520), followed by a triplex (addition of CD3 – Opal 570). All multiplex experiments were performed by repeating staining cycles in series, with microwave treatments between each cycle and at the end of the experiment. All multiplexed stains were finished with 4' 6-Diamidino-2-Phenylindole (DAPI) counterstaining. All stained slides were scanned using the Vectra automated quantitative pathology imaging system (Vectra 3.0.3; PerkinElmer) and analyzed using the InForm Advanced Image Analysis software (InForm 2.2.1; PerkinElmer). Multispectral images obtained by scanning were unmixed using spectral libraries built from images of single stained tissues for each reagent. Each cell was identified by detecting nuclear spectral element (DAPI) and the specific fluorescent spectra within every subcellular compartment was analyzed (CD20 – membrane, CD3 – cytoplasm, BCL2 – cytoplasm, MYC – nucleus). The fluorescent intensities for each marker were quantified on a per-pixel basis and were normalized between 0 – 100.
To analyze tumor-specific biomarker expression, tumor cells were selected using CD20 expression. All images were confirmed by pathologists (J.R and C.-S. P) for optimal staining by manual inspection. The tumor-specific quantitative immunofluorescence (QIF) score representing protein expression on a cell was calculated using the AQUA scoring method. The QIF scores for BCL2 and MYC were calculated as the signal intensities of each biomarker in the target compartment divided by the pixel area of the target compartment (Camp et al., 2002).

$$QIF\ score = \frac{\sum target\ intensity\ in\ compartment\ pixels}{\sum compartment\ pixel\ area}$$

2 SUPPLEMENTARY FIGURES AND TABLES

2.1 Supplementary Figures

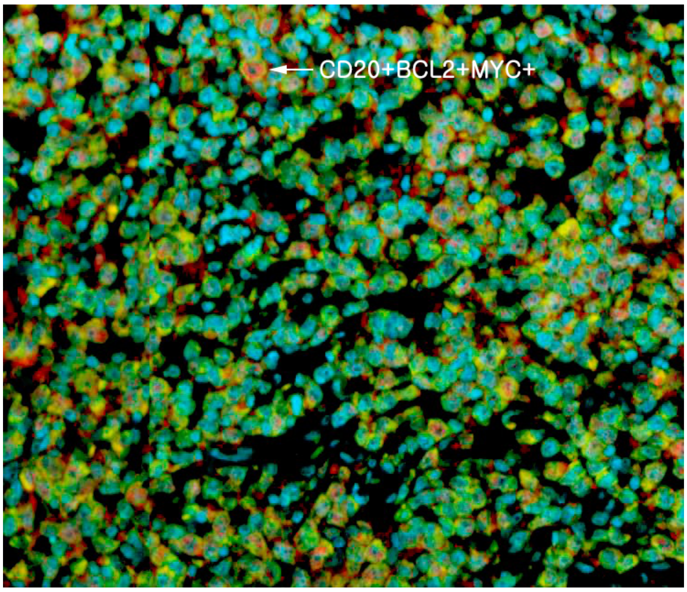

Figure S1: **Example of multiplex immunofluorescence image for quantitative immunofluorescence score.** Representative fluorescent multiplex images for CD20-Opal 650 (green), BCL2-Opal 520 (yellow), CD3-Opal 570 (orange), MYC-Opal 690 (red), and 4’ 6-Diamidino-2-Phenylindole (DAPI; blue). (Green: CD20+, Orange: CD3+, Yellow: BCL2+, Red: MYC+)

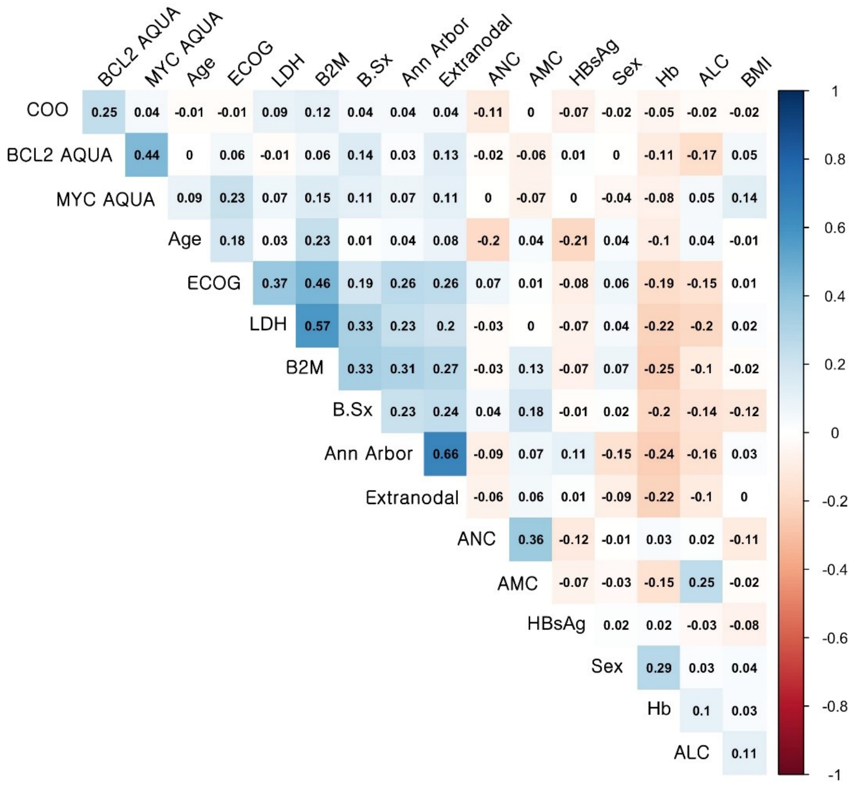

Figure S2: **Correlation matrix between covariates.** Correlation matrix shows correlation coefficients between covariates included in the analysis. Among the covariates, serum LDH level and serum B2M; Ann Arbor stage and presence of more than one extranodal site of disease shows positive correlation. (Abbreviation: COO, cell of origin; ECOG, Eastern Cooperative Oncology Group; LDH, lactate dehydrogenase; B2M, β2-microglobulin; ANC, absolute neutrophil count; AMC, absolute monocyte count; ALC, absolute lymphocyte count; Hb, hemoglobin)

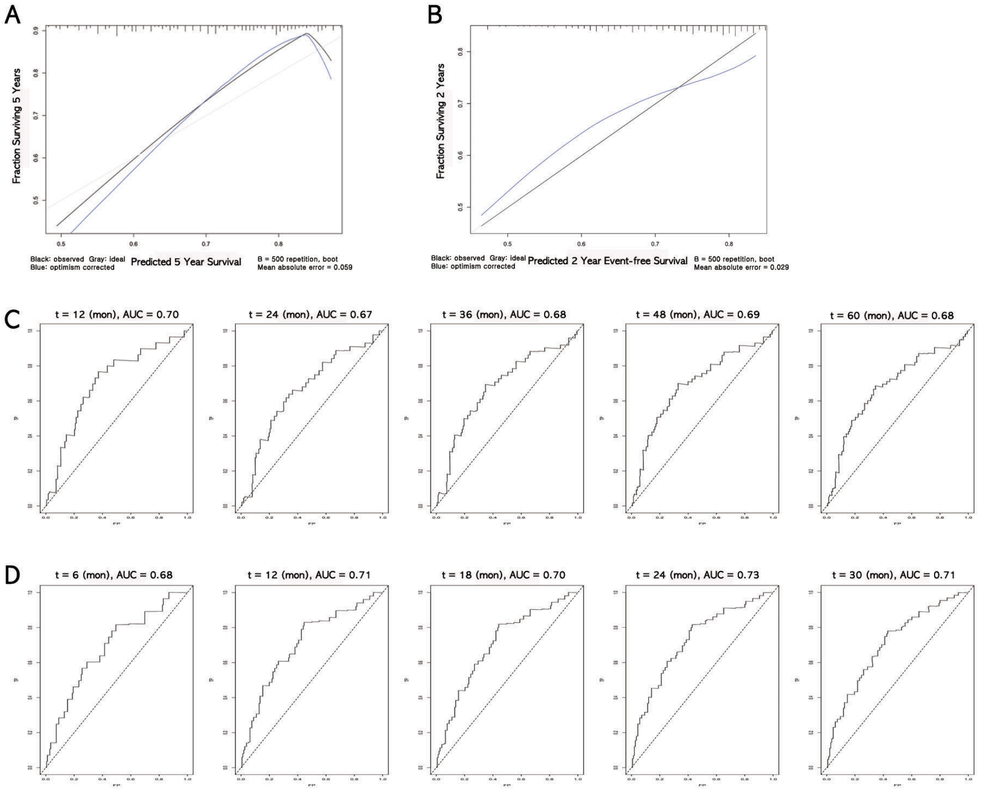

Figure S3: **Calibration plots and time-dependent receiver operating characteristic (ROC) curves** Calibration plots for **(A)** 5-year overall survival (OS) and **(B)** 2-year event-free survival (EFS) were plotted with 500 resampling. Black lines represent estimates of predicted vs. observed values and blue lines represent bias-corrected estimates. Time-dependend ROC curves for **(C)** 5-year OS and **(D)** 2-year EFS represents cumulative incidence by time (t).

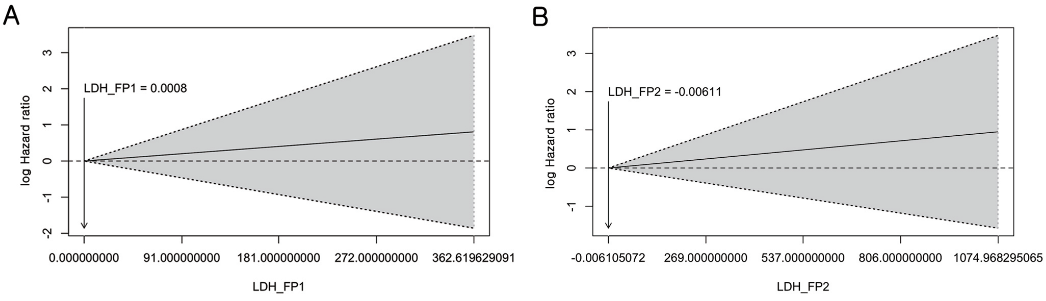

Figure S4: **Hazard ratio (HR) plots for each polynomial transformations of lactate dehydrogenase (LDH).** The HR plots shows that both FP1 transformed and FP2 transformed LDH is in linear relation to the log-hazard. The reference value is the minimum of the HR plot. (A) HR plot for LDH which are transformed as FP1. (B) HR plot for LDH which are transformed as FP2. (Abbreviation: FP, fractional polynomial)

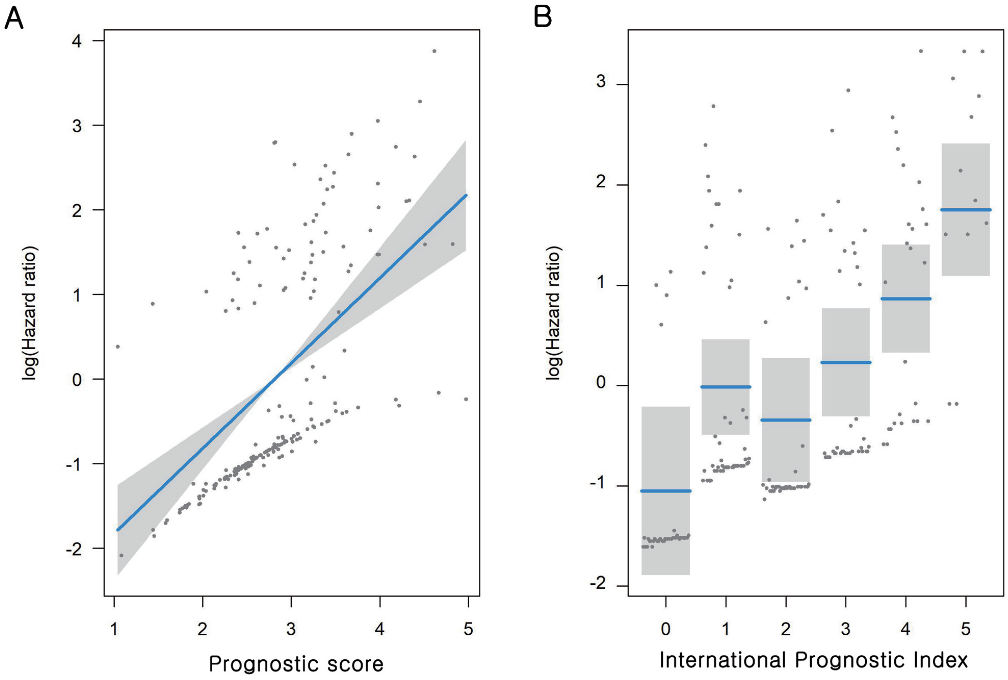

Figure S5: **Hazard ratio (HR) plots for (A) prognostic score and (B) International Prognostic Index (IPI).**

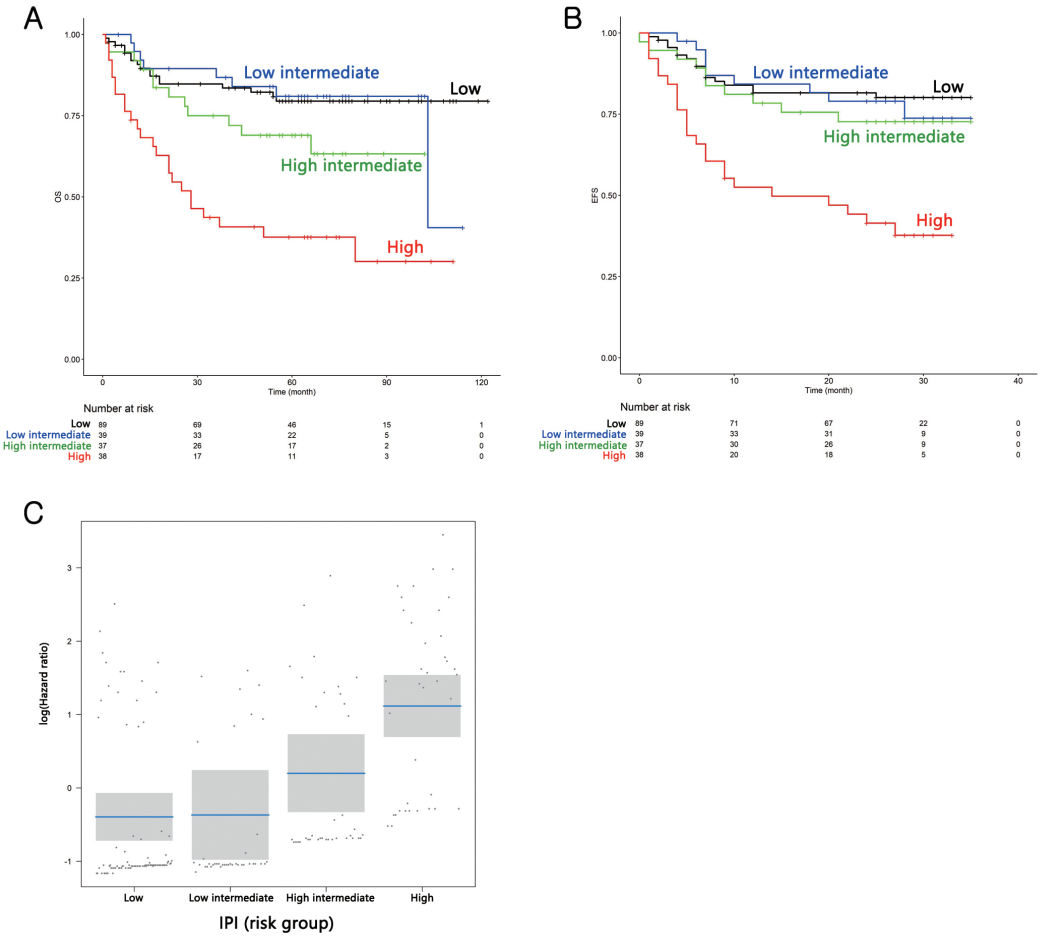

Figure S6: **Survival analysis according to the International prognostic index (IPI) risk group.** Kaplan–Meier survival curves for **(A)** overall survival (OS) and **(B)** 2-year event-free survival (EFS24) according to the IPI risk groups. **(C)** The HR plot for IPI risk groups.

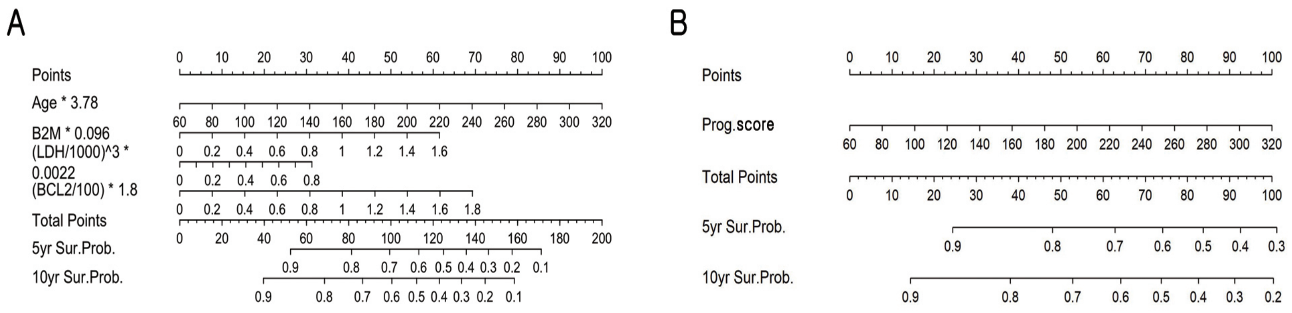

Figure S7: **Nomogram for overall survival.** Nomograms with **(A)** variables comprising the final prognostic model and **B** prognostic score calculated from the model.

2.2 Supplementary Table

Table S1. Inclusion frequencies for two correlated variables in 1,000 bootstrap replications.

	b2MG		Total
LDH	0	1	
0	87	288	375
1	120	463	583
Total	207	751	958

REFERENCES

Camp, R. L., Chung, G. G., and Rimm, D. L. (2002). Automated subcellular localization and quantification of protein expression in tissue microarrays. *Nat Med* 8, 1323–1327. doi:10.1038/nm791

TRIPOD Checklist: Prediction Model Development

Section/Topic	Item	Checklist Item	Page
**Title and abstract**			
Title	1	Identify the study as developing and/or validating a multivariable prediction model, the target population, and the outcome to be predicted.	1
Abstract	2	Provide a summary of objectives, study design, setting, participants, sample size, predictors, outcome, statistical analysis, results, and conclusions.	1
**Introduction**			
Background and objectives	3a	Explain the medical context (including whether diagnostic or prognostic) and rationale for developing or validating the multivariable prediction model, including references to existing models.	2
	3b	Specify the objectives, including whether the study describes the development or validation of the model or both.	3
**Methods**			
Source of data	4a	Describe the study design or source of data (e.g., randomized trial, cohort, or registry data), separately for the development and validation data sets, if applicable.	3
	4b	Specify the key study dates, including start of accrual; end of accrual; and, if applicable, end of follow-up.	3
Participants	5a	Specify key elements of the study setting (e.g., primary care, secondary care, general population) including number and location of centres.	3
	5b	Describe eligibility criteria for participants.	NA
	5c	Give details of treatments received, if relevant.	3
Outcome	6a	Clearly define the outcome that is predicted by the prediction model, including how and when assessed.	5
	6b	Report any actions to blind assessment of the outcome to be predicted.	NA
Predictors	7a	Clearly define all predictors used in developing or validating the multivariable prediction model, including how and when they were measured.	3 - 4
	7b	Report any actions to blind assessment of predictors for the outcome and other predictors.	NA
Sample size	8	Explain how the study size was arrived at.	3
Missing data	9	Describe how missing data were handled (e.g., complete-case analysis, single imputation, multiple imputation) with details of any imputation method.	3
Statistical analysis methods	10a	Describe how predictors were handled in the analyses.	
	10b	Specify type of model, all model-building procedures (including any predictor selection), and method for internal validation.	4
	10d	Specify all measures used to assess model performance and, if relevant, to compare multiple models.	4
Risk groups	11	Provide details on how risk groups were created, if done.	7
**Results**			
Participants	13a	Describe the flow of participants through the study, including the number of participants with and without the outcome and, if applicable, a summary of the follow-up time. A diagram may be helpful.	5
	13b	Describe the characteristics of the participants (basic demographics, clinical features, available predictors), including the number of participants with missing data for predictors and outcome.	Table 1
Model development	14a	Specify the number of participants and outcome events in each analysis.	5
	14b	If done, report the unadjusted association between each candidate predictor and outcome.	Table 1
Model specification	15a	Present the full prediction model to allow predictions for individuals (i.e., all regression coefficients, and model intercept or baseline survival at a given time point).	6
	15b	Explain how to the use the prediction model.	NA
Model performance	16	Report performance measures (with CIs) for the prediction model.	6
**Discussion**			
Limitations	18	Discuss any limitations of the study (such as nonrepresentative sample, few events per predictor, missing data).	8
Interpretation	19b	Give an overall interpretation of the results, considering objectives, limitations, and results from similar studies, and other relevant evidence.	7
Implications	20	Discuss the potential clinical use of the model and implications for future research.	8
**Other information**			
Supplementary information	21	Provide information about the availability of supplementary resources, such as study protocol, Web calculator, and data sets.	13
Funding	22	Give the source of funding and the role of the funders for the present study.	13

We recommend using the TRIPOD Checklist in conjunction with the TRIPOD Explanation and Elaboration document.
